# Performance evaluation of cementitious composites by designing an extrusion system for construction 3D printing

**DOI:** 10.1038/s41598-025-02860-9

**Published:** 2025-05-21

**Authors:** Shivashankarayya Hiremath, Gururaj Shrishail Mathapati, Dundesh S. Chiniwar, H. M. Vishwanatha

**Affiliations:** 1 Department of Mechatronics, Manipal Institute of Technology, Manipal Academy of Higher Education, Manipal, Karnataka 576104 India; 2 Survivability Signal Intelligence Research Center, Hanyang University, Seongdong-gu, Seoul, 04763 Korea; 3 Freshot Robotics Pvt. Ltd., Bangalore, Karnataka 560076 India; 4 Department of Mechanical and Industrial Engineering, Manipal Institute of Technology, Manipal Academy of Higher Education, Manipal, 576104 India

**Keywords:** Construction 3D printer, Extrusion system, Cementitious composite, Slump height, Computer-aided design, Infrastructure, Mechanical engineering, Civil engineering

## Abstract

The advancement of construction 3D printing (C3DP) relies heavily on optimizing the material formulation and hardware configuration. This study reports the development of a customized extrusion system—comprising a hopper, auger, and interchangeable nozzles— designed for use with locally sourced, cost-effective cementitious composites. A series of composite mixes were evaluated, with the CSSAW mix (cement, fine sand, fine soil, admixture, and water) exhibiting superior printability, achieving a slump height of 27 cm after 30 min and maintaining structural integrity across 11 printed layers. Compared to the baseline CSW mix, CSSAW showed a 92.8% improvement in flowability and a threefold enhancement in shape retention. Optimization of printing parameters, including a speed of 25 mm/s and layer height of 10 mm, further improved interlayer adhesion and surface quality. The rectangular nozzle was found to enhance deposition consistency and print stability. The findings establish a practical framework for deploying C3DP in real-world construction setups using sustainable materials and scalable extrusion systems.

## Introduction

 Extrusion-based 3D printing is a rapidly growing technology in the construction industry^1^. The technology has achieved significant milestones, enabling the printing of complex structures in less time and at a lower cost^2^. Extrusion-based printing, commonly referred to as 3D printing, creates structures by adding material layer by layer from a digital 3D model according to the desired design^3^. This additive approach offers distinct advantages over subtractive manufacturing processes, primarily by minimizing material waste and simplifying the manufacturing process^4^. As a result, additive manufacturing (AM) has revolutionized traditional manufacturing methods by strategically adding material. According to ISO/ASTM 52900-15 standards, AM technology is classified into seven main categories: material extrusion, binder jetting, direct energy deposition, material jetting, vat polymerization, sheet lamination, and powder bed fusion^5^. Each of these processes has varying levels of complexity, operating principles, and impacts on the quality and physical properties of the final product, as well as production costs^6^. The selection of a 3D printing method depends on the material used and the intended application, which imposes specific requirements^7^. For instance, materials such as metals, polymers, ceramics, and composites can be utilized in different AM methods depending on their applications, such as in construction^8^, automotive^9^, aerospace^10^, industrial products^11^, biomedical components^12^, and customizable consumer products^13^. This versatility highlights the adaptability and potential of AM across diverse industries^14^.

Construction 3D Printing (C3DP) is one of the most rapidly advancing technologies in the construction industry due to its ability to quickly assemble structures, minimize material waste, and handle complex designs^15^. As a result, the construction industry is increasingly adopting large-scale extrusion-based 3D printers with diverse techniques and material approaches^16^. Further, extrusion-based AM has gained significant popularity in recent years due to advancements in scaling up 3D printing technology^17^. This method utilizes gantry mechanisms for the direct deposition of material layer-by-layer to create additive structures^18^. A gantry mechanism in C3DP serves as a large, frame-like structure that supports and guides the movement of the 3D printer nozzle^19^. Using such a gantry, the deposition of construction materials, such as concrete, is controlled precisely^20^. It typically allows multi-axis movement (X, Y, and Z axes) of the print head or extruder, enabling accurate positioning and material deposition over a large build area, such as for houses or infrastructure components^21^. In C3DP, various extrusion machines are employed, including robotic systems with articulated robots, delta printers, gantry-type systems, and crane-type setups^22^. These systems facilitate layer-by-layer deposition of cementitious materials within the designated build area, making them well-suited for large-scale construction work^23^. This versatility highlights the adaptability and growing potential of C3DP in modern construction practices^14^.

In recent times, researchers have increasingly adopted C3DP, and the printed structures’ strength, stability, and performance have been analyzed^24^. For instance, Junhong Ye et al. utilized 3D printing to fabricate lightweight slabs of cementitious composites by creating hollow sections and investigated the flexural strength of concrete printed structures^25^. Similarly, Xiaoyan Sun et al. printed polyalcohol fibers with cementitious printing ink to study the stacking stability, durability, flowability, and load-bearing capacity of 3D-printed structures^26^. Several studies have highlighted the use of different concrete materials and varied mix ratios for construction purposes^27,28^. Kuznetsov D.V. et al. used gypsum and gypsum-cement binders to construct 3D-printed objects and evaluated the load-bearing capacity of the printed walls^29^. Zhihui Zhao et al. examined the synergistic effects of magnesium-to-phosphate mass ratio, borax content, and fly ash content on the initial setting time and rheological properties of 3D-printed magnesium potassium phosphate cement composites, focusing on extrudability and buildability^30^. Additionally, Rajeev Ranjan et al. summarized various materials used in 3D printing, including metals and alloys, ceramics, polymers, composites, smart materials, concrete, and biomaterials, emphasizing their utility in construction 3D printing^31^. The design of critical components in 3D printing machines, such as the hopper, nozzle, and auger, plays a crucial role in construction applications^32^. Albar et al. have explored modifications to extruder designs and investigated their impact on the printing performance of cementitious materials. Such designs contribute to maintaining shape retention and improving material flowability^33^. Joaquim Manoel Justino Netto et al. employed a two-screw configuration to evaluate the response of the extrusion unit in terms of flow and mixing performance^34^. Furthermore, printing parameters significantly influence the quality of the final printed product. Bing Lu et al. focused on the effects of printing parameters such as pumping rate, air injection pressure, nozzle travel speed, and nozzle standoff distance in construction 3D printing^35^. Guangchao Ji et al. studied the impact of nozzle orientation and scraper configurations on the buildability, mechanical properties, and microstructure of 3D-printed concrete components^36^. Recent literature reviews highlight the transformative potential of 3D concrete printing (3DCP) in construction. This technology offers advantages such as reduced costs, improved efficiency, and enhanced design flexibility^37^ 3DCP enables the creation of complex structural elements and integration of functional components, presenting opportunities for large-scale implementation^38^. The life cycle assessments indicate that 3DCP can significantly lower global warming potential compared to traditional construction methods. Future research should address these issues and focus on improving energy efficiency and optimizing structural designs to further enhance environmental performance^39^.

Recent studies on cementitious materials for 3D printing applications have explored alternative binders and additives to improve sustainability and performance. Geopolymers, alkali-activated systems, and MgO-based cements are being investigated as substitutes for traditional Portland cement^40^. The incorporation of supplementary cementitious materials and recycled wastes can reduce environmental impacts^41^. Clay soil has been proposed as an additive to partially replace cement, potentially reducing costs and improving printability with minimal strength loss^42^. Key properties for 3D-printed cementitious materials include flowability, extrudability, buildability, and open time in the fresh state, as well as density, strength, and shrinkage in the hardened state^43^. Researchers also explore structural optimization and energy-efficient printing systems to enhance environmental performance. Even though construction 3D printing has become a game-changing technology, several restrictions remain. Standard concrete mixtures have been the subject of numerous studies that have not optimized materials for additive manufacturing. Because of this, 3D printable materials frequently have inconsistent rheological characteristics, setting times, and strength development. There are presently no standardized testing methods for C3DP, and full-scale structural validation is still poorly understood. Moreover, the incorporation of high-voltage, plumbing, and electrical utilities into 3D printed structures has not received much attention. The performance of printed buildings in a variety of climatic settings, including high humidity, scorching deserts, and cold, is also not well documented. Furthermore, a lot of creative ideas are limited by material and structural constraints, underscoring the continuous difficulty of closing the gap. In short, the performance of C3DP depends not only on the concrete materials used but also on the printing parameters and extrusion design. Although significant progress has been made, further research is required to develop high-strength, high-quality, and durable 3D-printed construction products.

In the present study, a unique approach to optimizing the construction 3D printing (C3DP) process was implemented by reengineering critical extruder components, including a redesigned hopper, auger, and interchangeable nozzle geometries. These innovations were integrated into a custom-built extrusion system and systematically tested for their influence on 3D printability. A series of cementitious composite mixes, incorporating fine soil as a sustainable additive, were developed and evaluated based on their flowability and shape retention. In addition to material formulation, key printing parameters such as gantry speed, standoff layer height, and nozzle shape were fine-tuned to achieve optimal layer deposition and structural stability. Under these optimized conditions, a cavity wall structure was successfully printed, demonstrating the system’s capability to fabricate functional components with improved accuracy and sustainability. This work not only offers a practical, low-cost alternative for construction 3D printing using locally sourced materials but also lays the groundwork for the development of more adaptive and environmentally conscious design methodologies in future construction applications.

## Materials and methods

**Fig. 1:**
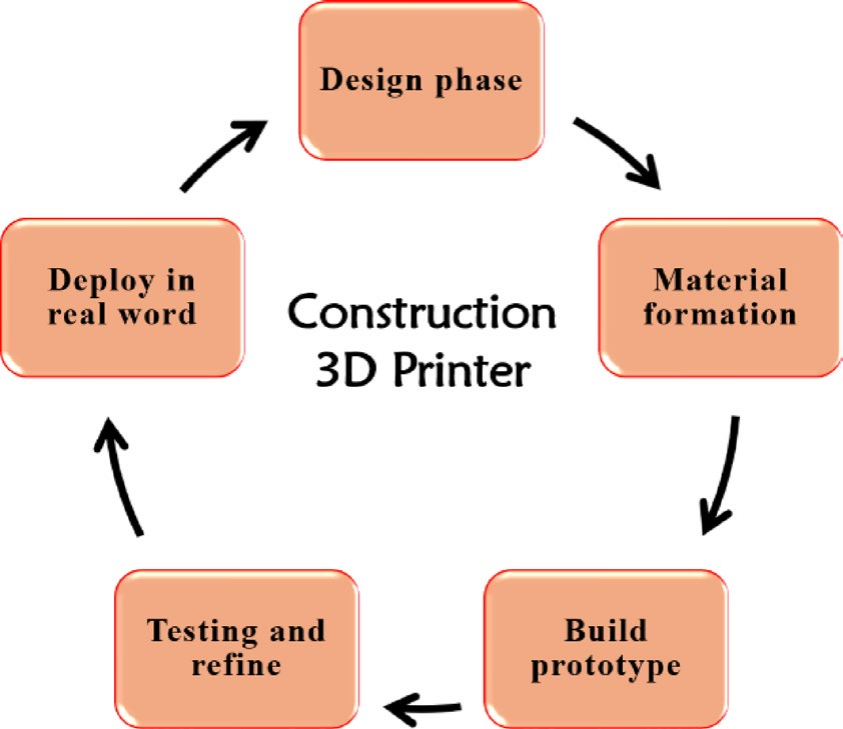
The phases of the large-format 3D printers.

The development of a large-scale 3D printer for producing pre-fabricated or on-site concrete structures is challenging^37^. It integrates an electromechanical system to control the material dispensers’ movement. An optimal method is needed for the proper mixing and extrusion of concrete slabs. The concrete mix must be specially formulated to be fluid enough for extrusion through a nozzle, yet stiff enough to maintain its shape after deposition. Admixtures are typically added to adjust the setting time, workability, and strength of the material. The 3D print steps followed to operate large-format 3D printers for concrete structures consist of a few major phases, shown in Fig. 1.

Initially, the 3D model of the structure is designed using Computer-aided design (CAD) software. The CAD model is then sliced into layers using software, followed by generating the path for the 3D printer nozzle to follow. Formulation of concrete mixture using different materials: cement, aggregates, admixtures, and additives like fibers or plasticizers is carried out. Further, the concrete 3D printer, which can be gantry-based, robotic arm-based, or delta-style, is calibrated for the print, including nozzle size, layer height, and print speed. The concrete is extruded layer by layer according to the predefined path. Precision control ensures that each layer bonds well with the previous one, maintaining structural integrity. Finally, the structure is printed layer-by-layer and allowed to cure. Depending on the mix and environmental conditions, additional treatments like moisture curing may be applied to ensure proper hydration and strength development, and then deployed in real-world applications.

### Design of extruder and nozzle

**Fig. 2:**
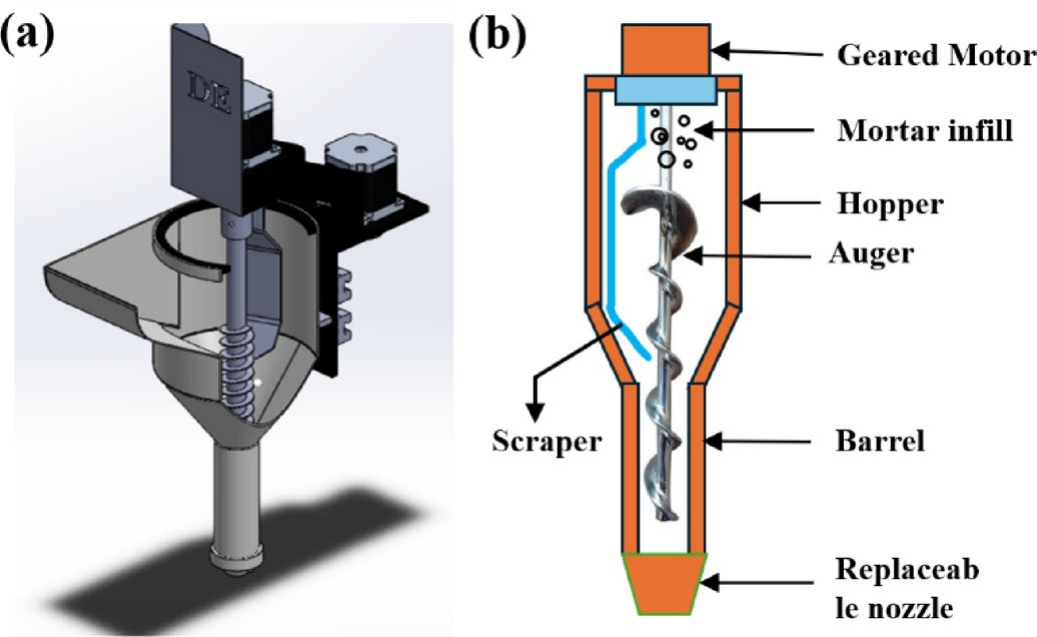
Extruder of C3DP: (**a**) CAD model of the extruder and (**b**) Components of the extruder.

The extruder system is a critical component of a C3DP, responsible for depositing the concrete mixture in precise layers to create the desired structure^38^. The design of this system must ensure efficient material flow, accurate deposition, and adequate bonding between layers, while also managing the unique challenges posed by concrete as a printing material. Also, the design of the extruder system to achieve results of repeated trial and error is based on the rheological characteristics, such as flowability and fluidity of cementitious-based materials. Figure 2 shows the CAD model and parts of the extruder system used in C3DP.

**Fig. 3:**
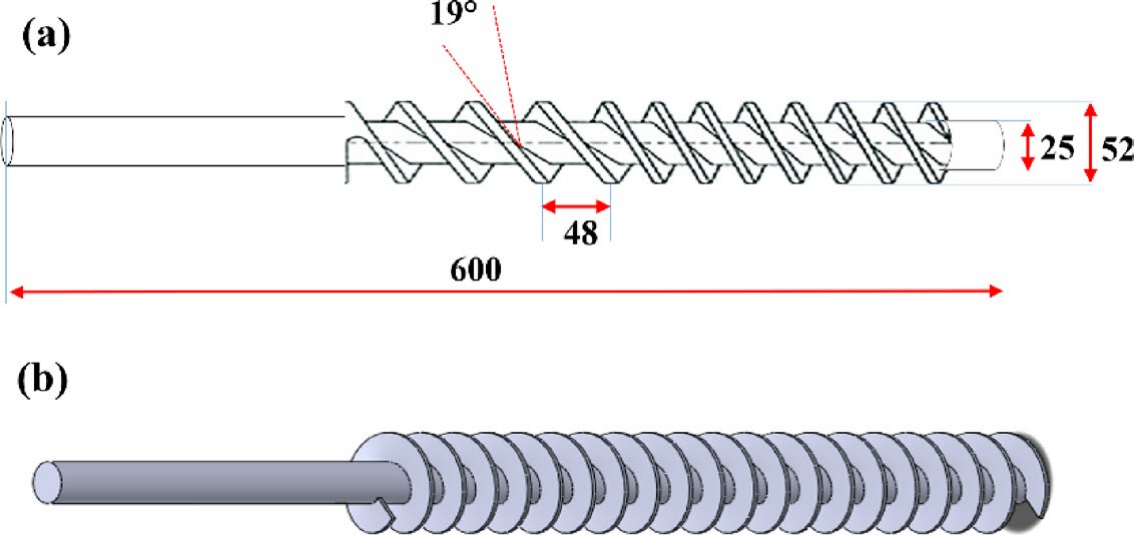
Auger screw: (**a**) dimensions and (**b**) CAD model.

The top container or hopper holds the concrete mix and feeds it into the extruder. The rotating auger mechanism ensures the concrete is pushed downward while uniformly mixing it with additives during extrusion. A geared motor controls the rate at which the concrete exits through the hopper to the nozzle, thereby maintaining consistency. The well-mixed concrete is then extruded through the nozzle and deposited. The entire system is enclosed within a barrel that provides structural support and protects the internal components from wear and environmental exposure. Further, the auger screw is an essential component in C3DP specifically in concrete extrusion systems. Its primary function is to facilitate the controlled flow and uniform distribution of concrete material from the hopper to the nozzle. The auger screw, with its helical design, effectively pushes the concrete mix downward. This mechanism ensures a consistent flow of material, which is required to keep the layers uniform during the printing process. The auger screw used in the extrusion system was designed with the following characteristics to ensure optimal performance and material flow. The overall length of the auger screw is 600 mm, allowing sufficient capacity for material transport and extrusion. A screw pitch of 48 mm is selected to provide an efficient balance between material feed rate and extrusion precision. The inner diameter and outer diameter of the auger screw are 25 mm and 52 mm respectively, ensuring compatibility with the extrusion system and effective material flow. The blade is set at a 19° angle to facilitate smooth material conveyance and uniform extrusion. The pitch-to-diameter ratio of 1.92 is implemented to optimize the extrusion under specific pressure conditions. This ratio is critical for maintaining consistency in the extrusion process, as it governs the relationship between the screw’s rotational speed and the material flow rate. The pitch-to-diameter ratio is directly proportional to the rotational pace of the screw, enabling precise control over the material output. Adjusting the extrusion factor ensures uniform layer buildup and consistency, which are essential for achieving high-quality construction in 3D printing. The auger screw dimensions and CAD design are shown in Fig. 3.

**Fig. 4:**
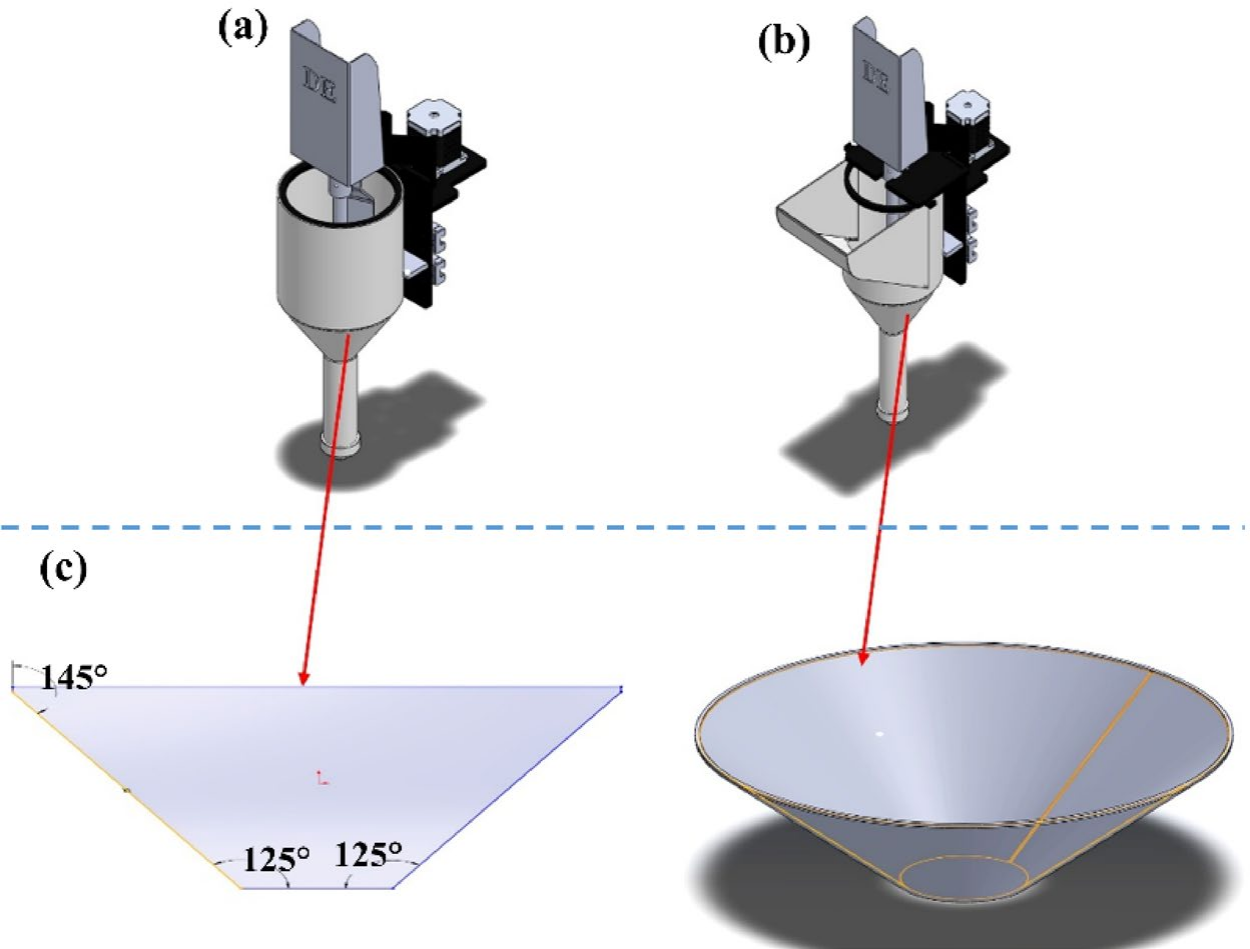
The hopper design: (**a**) Cylindrical shape, (**b**) Elongated shape, and (**c**) Inclination angles.

The hopper is another crucial component of the concrete extrusion system, responsible for holding the prepared concrete mix and feeding it uniformly into the auger screw. The design and shape of the hopper significantly influence the material flow rate, extrusion consistency, and overall system performance. The initial design of the hopper and the modified configurations are shown in Fig. 4**(a-c).**

The hopper is designed with adequate capacity to hold the required volume of concrete mix, ensuring uninterrupted operation during 3D construction. The walls of the hopper are angled to facilitate the smooth flow of the concrete mix into the auger, preventing clumping or blockages. To optimize material capacity for the nozzle head, the hopper funnel and angle of inclination were carefully designed. As shown **in** Fig. 4**(c)**, the angles were set to 145° at the top and 125° at the bottom of the funnel, ensuring a smooth flow of the mixture. Additionally, low-friction materials were used to minimize material sticking and ensure uniform feeding. The hopper’s shape has been further modified into two configurations— cylindrical and elongated, shown in Fig. 4**(a**,** b)**, respectively.

The cylindrical shape ensures a more uniform and continuous flow of material due to the absence of corners where concrete may accumulate. This design supports steady extrusion but requires careful monitoring to prevent stagnation. The elongated shape provides better stability and structural support due to its square sides and edges. This design can handle a larger volume of material and offers easier feeding into the extrusion system. Both designs ensure compatibility with the auger system and provide seamless feeding of the concrete mix, maintaining consistent extrusion during the 3D printing process.

The scraper in the concrete 3D printer is an essential component to ensure the extruded material’s uniform layering. Positioned near the nozzle, the scraper smooths and levels the deposited concrete, removing excess material to maintain precision in the printed structure. This contributes to achieving consistent layer thickness, improving the surface finish and structural integrity of the final print. Additionally, the scraper minimizes material waste and ensures seamless bonding between layers, enhancing the overall quality of the construction process. The overall barrel diameter of the hopper is 221 mm inner and the hopper entry is 360 mm which is selected based on the size of the screw used.

**Fig. 5:**
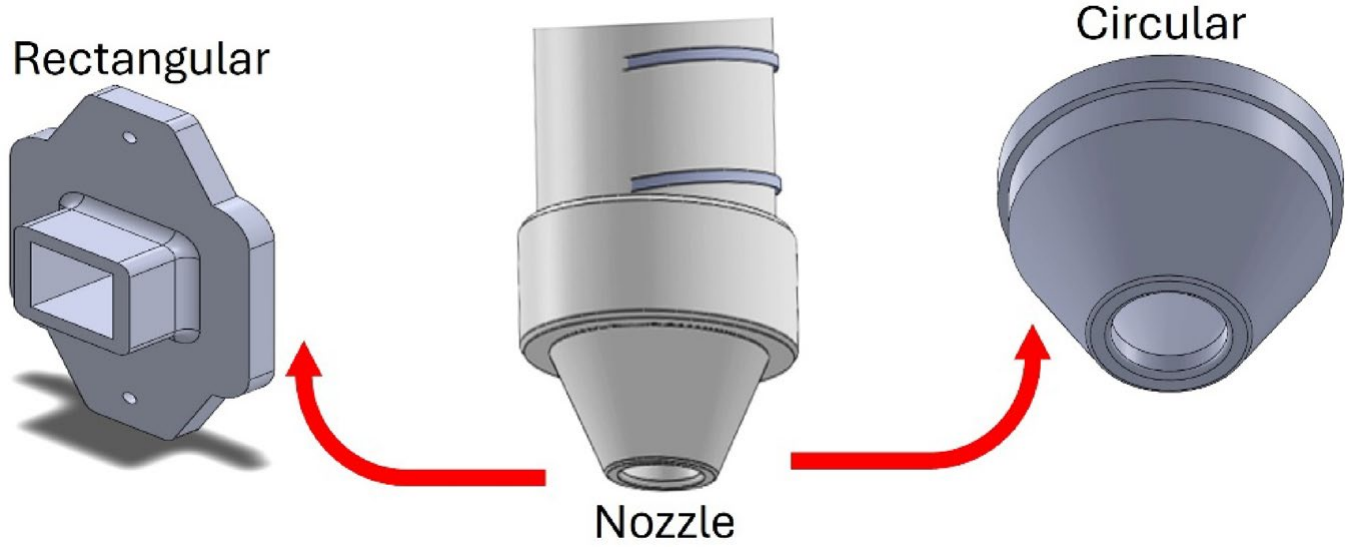
Rectangular and circular shapes of the nozzle.

The nozzle is an important component of the concrete 3D printing system, responsible for shaping and depositing the extruded concrete onto the desired surface. The nozzle’s design significantly influences the precision, flow rate, and overall quality of the printed structure. The chosen nozzle designs are rectangular and circular shapes, shown in Fig. 5. The shapes are chosen based on specific construction applications. These shapes ensure stability during extrusion and consistency in material flow. The circular-shaped nozzle facilitates continuous flow with minimal clogging and is particularly effective in producing cylindrical structures. The rectangular nozzle, on the other hand, is ideal for creating sharp edges and flat surfaces. Both nozzle shapes are engineered to ensure compatibility with the extruder system, enabling uniform material deposition and precise control throughout the C3DP process.

**Fig. 6:**
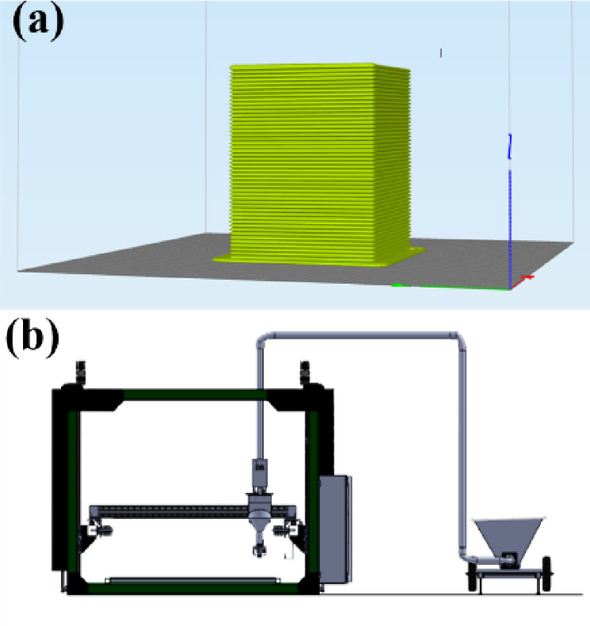
Virtual modes of: (**a**) print layer by layer, (**b**) construction 3D printer.

In the 3D design of construction, a variety of tools and interfaces are utilized to create, visualize, and manage complex structures. These tools are specifically designed to cater to architects, engineers, and contractors, streamlining the design and construction process. Initially, the required prototypes were designed using computer-aided design (CAD) software. The generated models were then imported into slicing software to control the print movement of the components. The nozzle’s path movements were adjusted using G-code, which provided precise commands to the machine. In the slicing software, various parameters such as moving speed, nozzle coordinates, orientation, and other settings can be programmed through a graphical user interface (GUI). These tools enable the design of complex and customized structures, followed by importing the files into slicing software for compatibility. The slicing software also simulates the print conditions in virtual mode, helping users verify the print quality and determine the need for support structures, if any, for building the components. Figures 6**(a) and (b)** illustrate the virtual mode of the 3D model, showcasing how it is printed layer-by-layer using a complete construction 3D printer. In this study, SolidWorks software was used to model the 3D parts due to its ease of use and ability to quickly build models. Subsequently, Simplify3D slicing software was employed, offering greater flexibility in adjusting key parameters such as gantry movement, layer standoff distance, print resolution, and extrusion factors.

### Cementitious materials for 3D printing

**Fig. 7:**
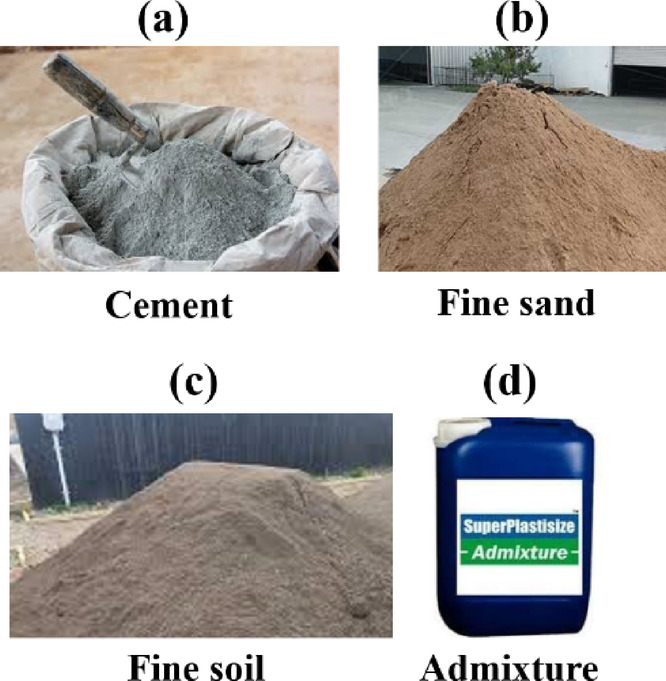
Cementitious materials used for 3D printing.

Cementitious-based additive materials have been used to enhance the strength and physical performance of ordinary cement-based composites. Cement alone poses environmental concerns, including the release of unwanted gases that contribute to global warming. Therefore, sustainable materials have been introduced as additives to improve the performance and environmental impact of cement, resulting in stronger, printable materials. In this study, materials such as cement, fine sand, fine soil, and admixtures, shown in Fig. 7, were used in various proportions to prepare cementitious composites.

Cement was used in the 3D printing construction process, and its Physico-mechanical and chemical properties are presented in Table 1.

**Table 1:** The detailed Physico-mechanical and chemical characteristics of the cement^44–46^.

Physical mechanical		Chemical composition	
Properties	Values	Group	Percentage(wt%)
Standard Consistency of Water (%)	28.4	CaO	51.42
Density/(g/cm^2^)	2.8–3.03	SiO_2_	24.99
Flexural Strength (MPa) 3–28 days	6.5–9.0	Al_2_O_3_	8.26
Compressive Strength(MPa) 3 to 28 days	29.5–58	Fe_2_O_3_	4.03
Specific Surface Area/(m^2^/kg)	365	MgO	3.71
		SO_3_	2.51

 For the construction process, sand with an approximate particle size of up to 4.75 mm was used. Fine aggregates, including sand, typically consist of particles that fall within a specific size distribution, commonly ranging from 4.75 mm to 1.18 mm and from 600 microns to 150 microns, as determined through sieve analysis. Similarly, fine soil was incorporated into the concrete mix, and its properties are detailed in Table 2^47–50^.

**Table 2:** The properties of sand and fine soil.

Properties	Particle Size (mm)	Max Dry Density (g/cm³)	Specific Gravity	Fineness Modulus	Absorption Capacity (%)	Moisture Content (%)
Fine Sand	1.88–4.70	1.52–1.60	2.5–3.0	1.2–3.2	< 2%	2–6
Fine Soil	0.0050–0.0075	1.78	2.46–2.71	–	–	15–19.2

In this study, a liquid-based Polycarboxylate Ether superplasticizer was used for construction 3D printing. This type of slump-retaining superplasticizer contains ester groups that slowly hydrolyze in the alkaline environment of cement, gradually releasing anchoring groups. Slump loss in concrete is primarily due to the decreasing concentration of superplasticizer in the cement paste. As the concentration drops, so does the paste’s fluidity. Since ordinary Portland cement has an initial setting time of around 3 h, a synergistic combination of a retarder and PCE is essential for prolonged workability. The admixture was added to a slag-containing mix (approximately 15 ml). The key properties of the admixture are provided in Table 3.

**Table 3:** Properties of polycarboxylate ether superplasticizer.

Item	Standard
Appearance	Light yellow to transparent liquid
Solid Content	50 ± 1%
pH	3–5
Viscosity	Max 1100 cPs
Specific Gravity	1.09 ± 0.02
Dosage	0.5–2.0% of the binder material

The mixing ratios and formulations were designed to evaluate the compatibility of the mixtures for extrusion and 3D printing. The specific ratios were used for each formulation. Three material configurations were tested across three mix trials: CSW, CSSW, and CSSAW. The CSW is a combination of cement, fine sand, and water. The CSSW is a combination of cement, fine sand, fine soil, and water. The CSSAW is a combination of cement, fine sand, fine soil, admixture, and water. The detailed proportions of these mixes are given in Table 4.

**Table 4:** Mix ratio used in the formulation of cementitious composite.

Mixture code	Cement (kg)	Fine Sand (kg)	Fine Soil (kg)	Admixtures (ml)	Water (ltr)
CSW	3	6	-	-	2
CSSW	4	5	1	-	1
CSSAW	5	4	2	15	1.5

The mixing procedure was designed to ensure a homogeneous blend of materials suitable for extrusion-based construction 3D printing. The CSSAW cementitious composite was created using a clean mixing container, with cement, fine sand, and fine soil added in their dry states. These dry components were mixed thoroughly for about 2–3 min to ensure even distribution of fine particles and avoid clumping during wet mixing. Approximately 80% of the total water was slowly added while the mixer was running. This helped initiate uniform hydration and maintain the workability required for 3D printing. The polycarboxylate ether-based superplasticizer was diluted in a small portion of water before being added gradually. The remaining water was added after the admixture, maintaining the desired water-to-cement ratio. All components were mixed thoroughly for an additional 2–3 min to achieve a homogeneous, lump-free paste suitable for extrusion.

Furthermore, the cementitious composite samples were evaluated for flowability before the printing process. The slump test was conducted to determine how easily a fresh concrete mix would flow and to assess its consistency. The test involves filling a cone with cementitious composite and then lifting it vertically to measure the height difference between the concrete and the cone. This height difference is referred to as the slump height. Similarly, a shape retention test was conducted to evaluate the designed extruder’s capability to print a wide range of cementitious composites concerning flowability, open time, and setting time. Layers of composite paste were printed successively, and the printed samples were allowed to set for 50–60 min. After the setting process, the visual appearance of the composite samples was carefully examined.

### Construction 3D printer

**Fig. 8:**
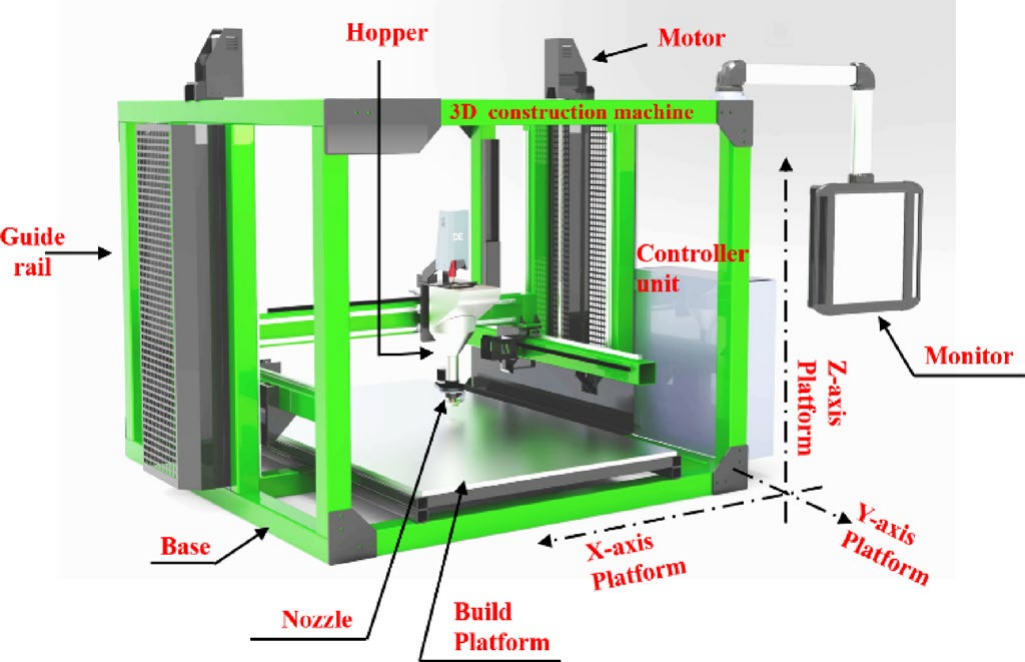
Large area C3DP machine.

Construction 3D printers are advanced machines designed to automate the building process by layering materials, such as cementitious composites, to create structures. These printers utilize additive manufacturing technology to construct walls, foundations, and even entire buildings with precision and efficiency. The complete 3D construction machine with a gantry mechanism is shown in Fig. 8. In this study, the construction 3D printer was used to test the performance of cementitious composites by varying the print conditions— printer speed, layer standoff height, and extrusion ratio. The layer thickness during printing was adjusted by modifying the nozzle settings, the speed of gantry movement was varied, and the extrusion ratio of the composite was altered to achieve accurate layers with high-performance construction. These parameters were systematically varied, and their influence on the printed structure was analyzed.

## Results and discussions

### Extruder and nozzle

**Fig. 9:**
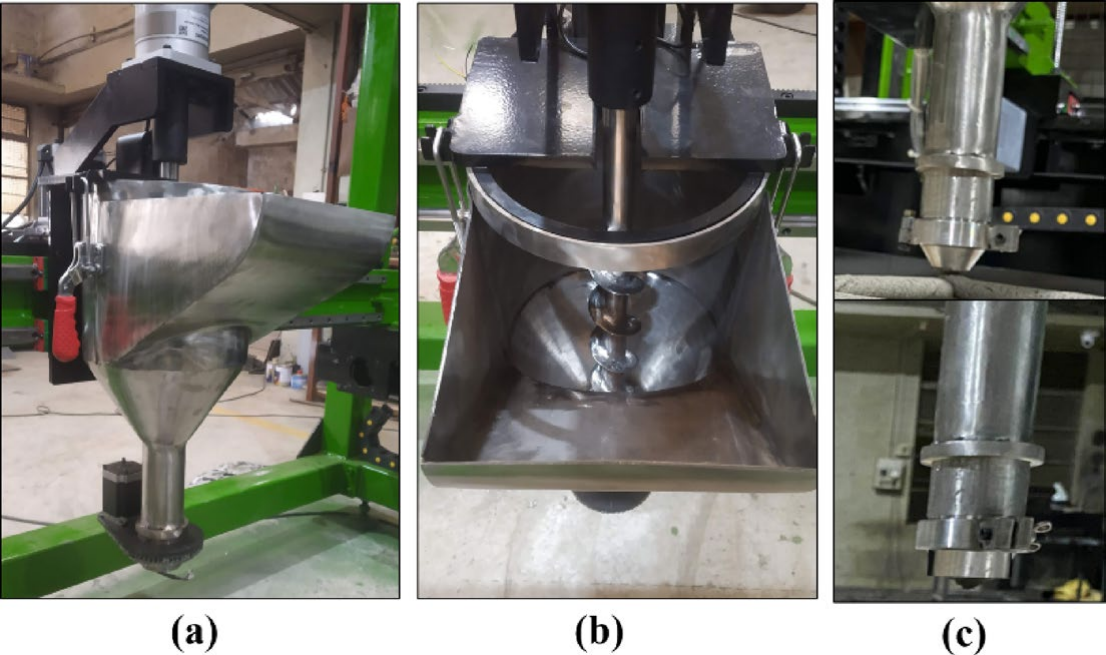
Fabricated extruder part (**a**) Elongated shape hopper, (**b**) Assembled Auger, (**c**) circular and rectangular nozzle.

The extruder was initially fabricated using stainless steel, chosen for its resistance to rust, smooth material flow, and protection against environmental degradation. The fully assembled extruder, comprising the hopper, auger, and nozzle, is shown in Fig. 9. It was observed that all extrusion components were successfully fabricated and assembled onto the 3D construction machine. The components were perfectly aligned with the design specifications and performed effectively as part of the assembled unit during the printing process.

### Properties of cementitious composite

**Fig. 10:**
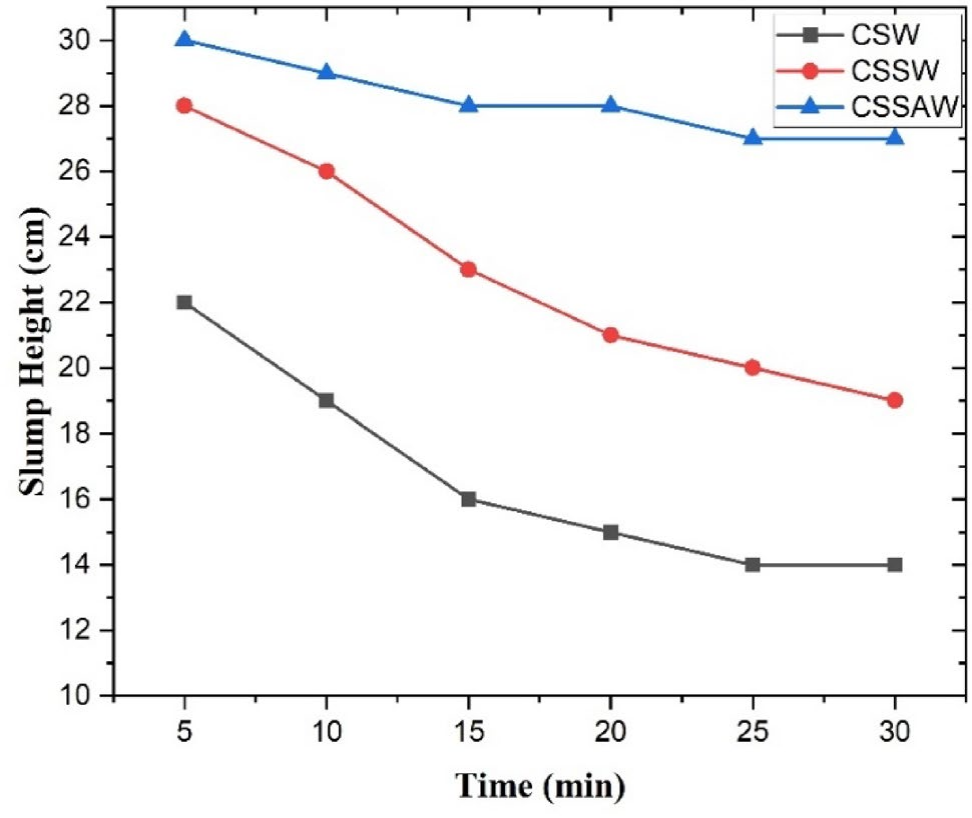
Slump height of different cementitious composite materials.

The printability of various cementitious composites was tested under different printing conditions, focusing on flowability and the shape retention of the printed material. The slump data for all three mixes recorded over time are presented in Fig. 10. It is observed that the slump height of all three cementitious composites decreased as time increased. In particular, the CSW composite showed unsatisfactory performance, as the slump height gradually reduced, likely due to the higher water content in the mix ratio. Furthermore, the addition of fine soil to the CSW mix enhanced flowability compared to CSW, but not to the desired performance criteria. To address this issue, a superplasticizer was added as an admixture to the CSSW composite to form a CSSAW composite. It is noted that including the superplasticizer significantly improved flowability, bringing it closer to the expected requirements for 3D printing applications.

**Fig. 11:**
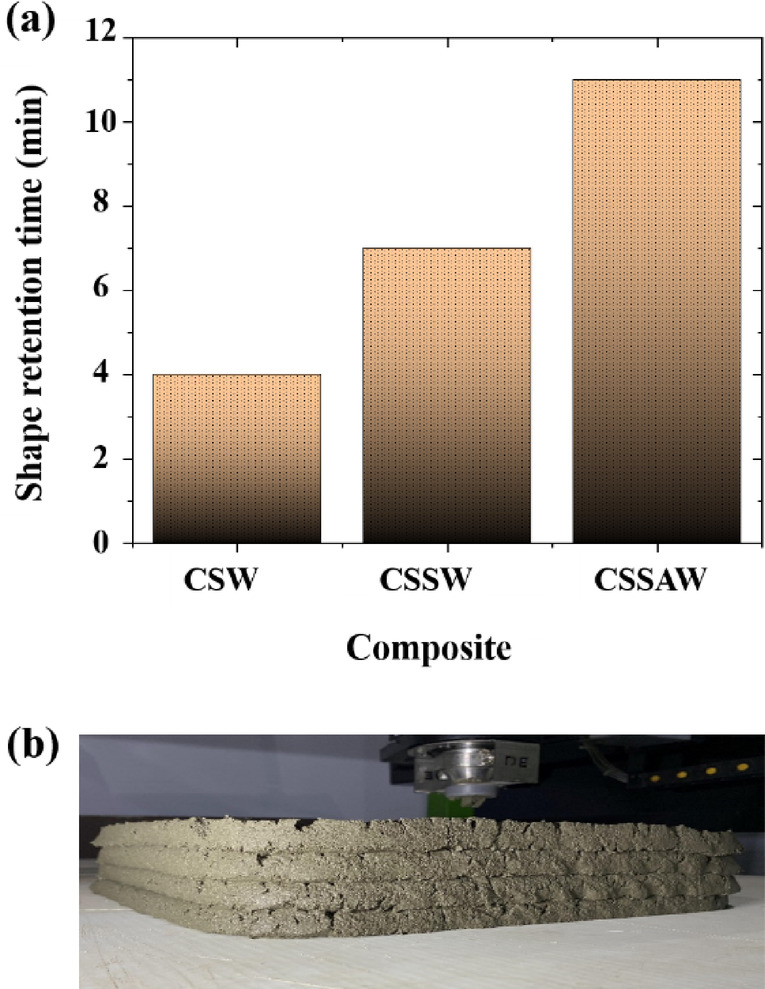
(**a**) Shape retention of the cementitious composite (**b**) Image of shape retention test of CSSW sample.

In the next phase, the shape retention test was conducted for all three cementitious composites by printing four layers and visually inspecting the time the printed composites took to retain their shape. Figure 11 illustrates the shape retention performance of the cementitious composites, along with the printed image of the CSSW sample. It is observed that the CSSAW composite could retain its shape for a longer time as compared to the other two composites. Specifically, the CSSAW sample retained its shape for approximately 11 min before gradually flattening under the weight of subsequent layers. Hence, the CSSAW, admixture-enhanced composite, demonstrated superior flowability and shape retention performance, making it a more suitable material for the construction of 3D printing parts. Considering the better performance of the CSSAW composite, the printing of the composite using the extruder was carried out using the CSSAW composite.

### Effect of extruder speed and layer height

**Fig. 12:**
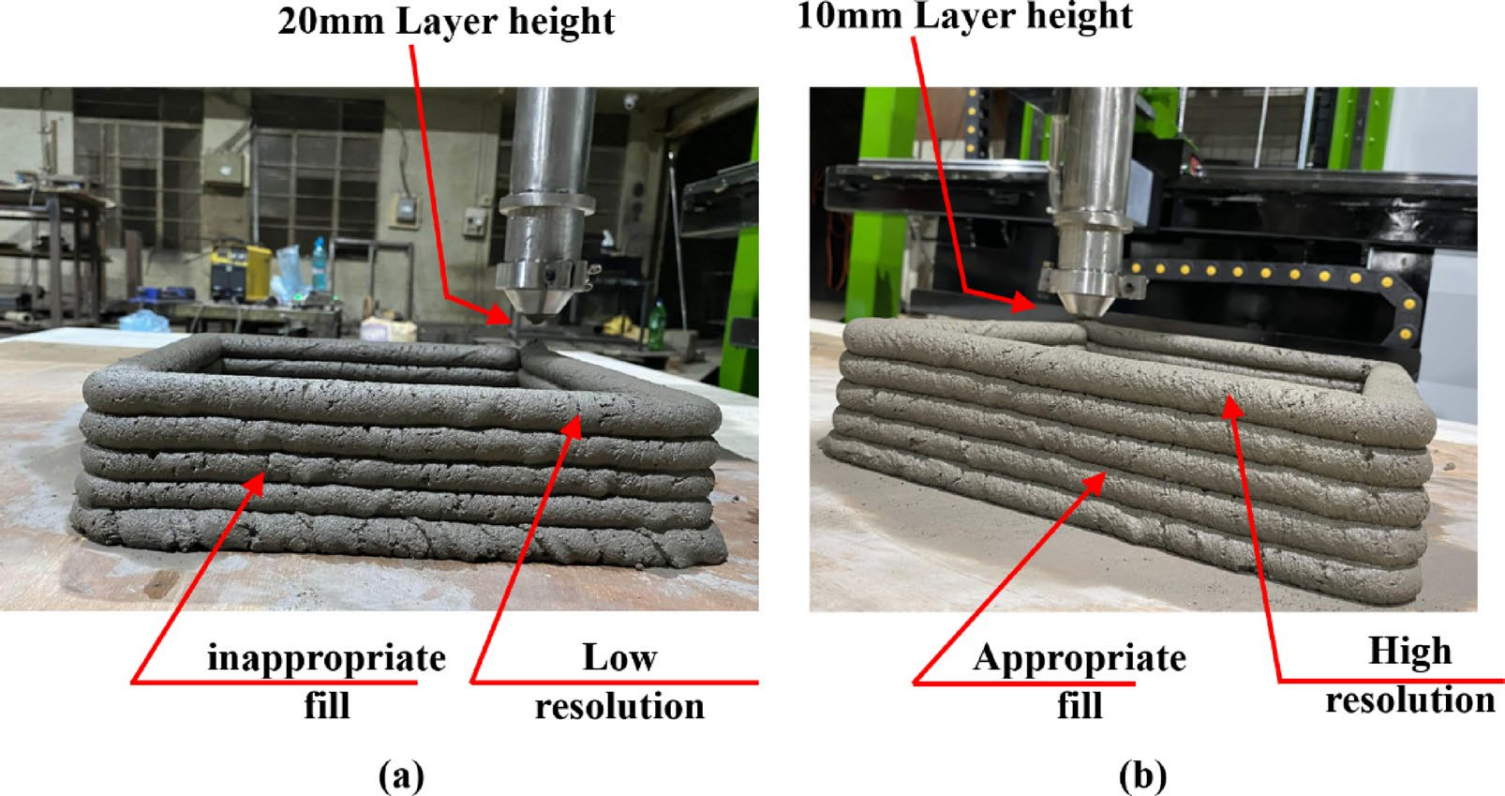
3D printed part at varied print conditions of extruder speed and layer height: (**a**) at 30 mm/s and 20 mm, (**b**) at 25 mm/s and 10 mm.

Further, the CSSAW composite was used to print samples under varied printing conditions, including printer speed, layer stand-off height, print resolution, and extrusion factor. Initially, the gantry or extruder speed was set to 30 mm/s, the stack layer height to 20 mm, and the extrusion factor to 40%; the print quality was then observed. Figures 12**(a) and (b)** show the differences in the printed 3D parts under these varied conditions. In the initial setup, the printed parts exhibited lower resolution and incomplete layer filling, which is possibly due to the higher speed and greater extrusion height. The reduced speed and layer height of 25 mm/s and 10 mm, respectively, are found to significantly improve the performance and print quality of the built parts. Hence, it is critical to regulate the gantry speed and appropriately adjust the layer height to achieve better-quality prints using the extrusion-based 3D printing system.

### Effect of the shape of the nozzle

**Fig. 13:**
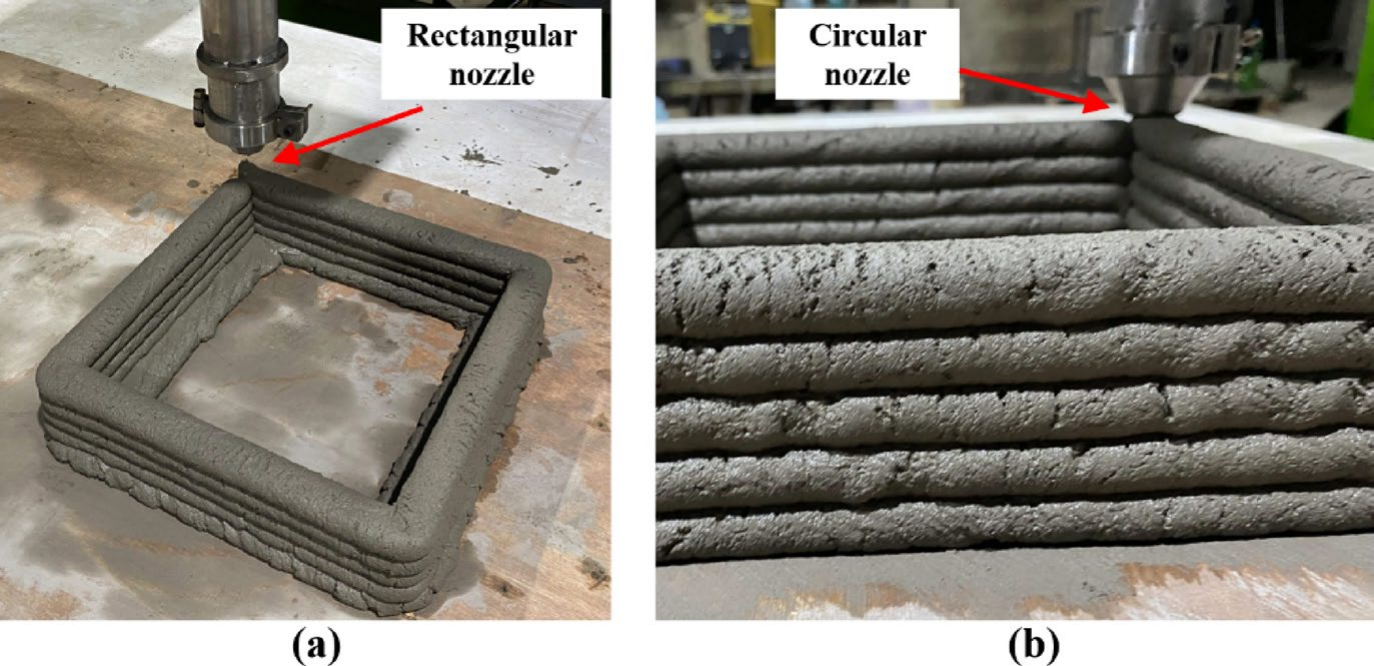
The shape of the rectangular and circular nozzle affects in C3DP.

The extrusion machine was used to print layer by layer using the two different nozzles. The effect of the shape of the nozzle was analyzed to understand the layer collapsibility during the printing. Figures 13**(a) and (b)** illustrate the layered structures printed using rectangular and circular-shaped nozzles, respectively. It is observed that composite layers printed using the rectangular nozzle resulted in flattened layers with proper bonding between successive layers. In contrast, the circular nozzle resulted in layers with visible radial edges and notches, which introduced stress concentrations at the layer interfaces.

**Fig. 14:**
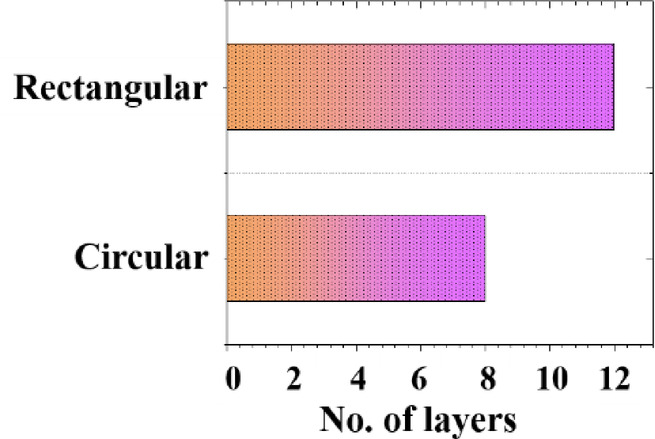
Effect of the nozzle on collapsibility of the 3D printed part.

The collapsibility of the printed parts from both nozzle shapes was visually examined, and the results are shown in Fig. 14. The results indicated that the rectangular nozzle provided greater stability with an increased number of layers. Specifically, the rectangular nozzle was able to print up to 12 layers before collapsing, whereas the circular nozzle could only achieve a maximum of 8 layers. This reduced stability with the circular nozzle may be attributed to improper bonding between the layers. Therefore, the rectangular-shaped nozzle exhibited superior stability and bonding performance, making it more suitable for layer-by-layer printing of cementitious composites. These findings highlight the critical role of nozzle shape in 3D printing applications involving cementitious materials.

**Fig. 15:**
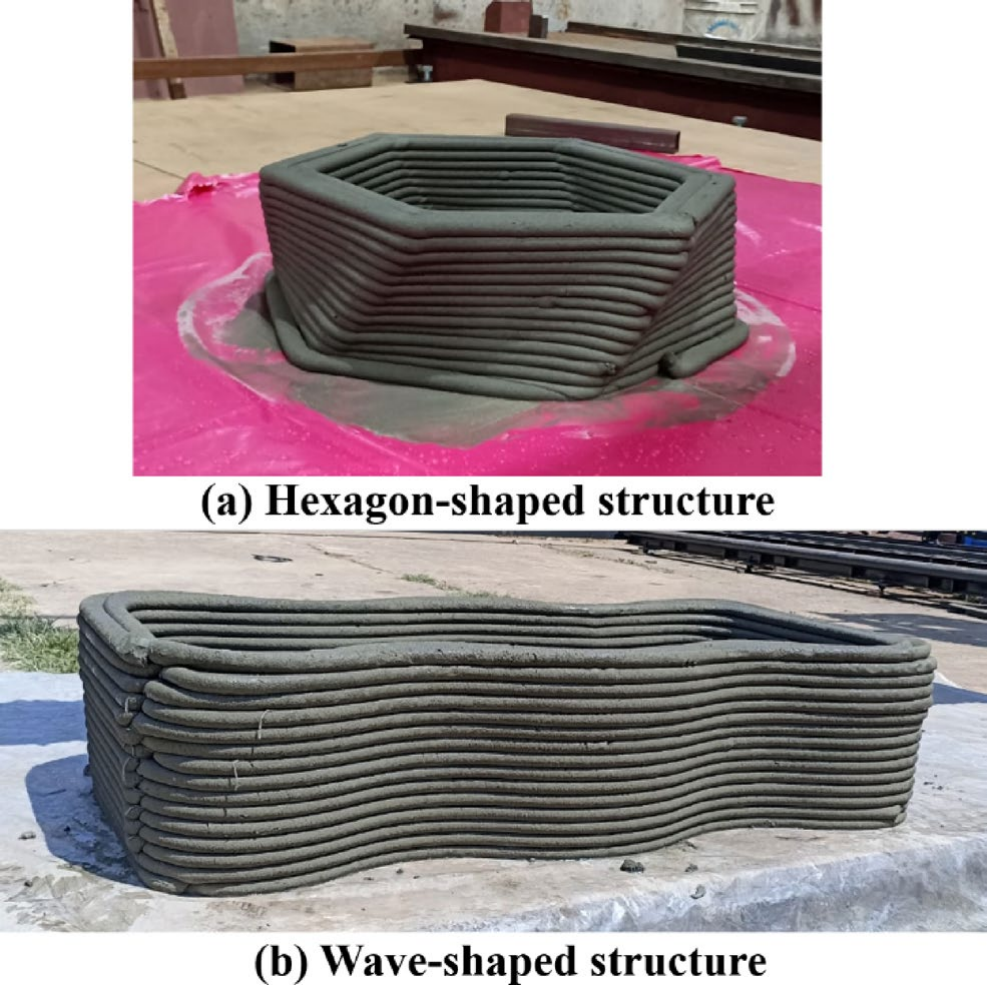
Prototype of C3DP of (**a**) Hexagon-shaped structure, (**b**) Wave-shaped structure.

At the end of the study, after multiple trials to optimize the flowability, shape retention, printing conditions, and nozzle design of the extruder machine, the printability and functionality of the 3D-printed construction material were successfully demonstrated. Two designs were printed using the cementitious composite: a slant angle with a hexagon-shaped and a tub with wave-shaped structures, as shown in Figs. 15**(a) and (b)**, respectively. The final printed prototypes of the hexagon and wave structures showcased a solid platform, reflecting improvements in 3D construction printing achieved by optimizing various parameters. The results demonstrate that multiple errors were resolved, leading to a properly printed block and the ability to print different shapes more efficiently. The printed structures were inspected and found to possess excellent strength, durability, and well-laminated layers. These findings validate the proposed 3D printer design and the optimized printability of the cementitious composite, highlighting the printer’s capability to produce high-quality construction materials. The materials can be tailored to specific designs, offering faster, cost-effective, and long-lasting solutions for construction applications.

## Conclusion

This study demonstrated the successful development of a cost-effective extrusion system tailored for construction 3D printing, comprising a hopper, auger, and modular nozzle designs. Among the three tested cementitious composite mixes, the CSSAW mix (cement, fine sand, fine soil, admixture, and water) showed superior workability, achieving a slump height of 27 cm after 30 min, and excellent shape retention during printing. The rectangular nozzle enabled stable deposition of up to 11 layers without structural collapse, with optimal print settings (25 mm/s speed and 10 mm layer height) significantly enhancing build quality and interlayer bonding. The system also showed adaptability in printing different prototype structures, confirming its potential for rapid and sustainable construction applications. The use of locally available materials and simple mechanical components makes this approach especially promising for low-resource or remote environments. Future work would focus on optimizing material formulations and extrusion mechanisms for improved performance and reliability.

## Data Availability

All data generated or analysed during this study are included in this published article.
